# Port site closure after laparoscopic surgery

**DOI:** 10.4103/0972-9941.62534

**Published:** 2010

**Authors:** P R Shah, N Naguib, K Thippeswammy, A G Masoud

**Affiliations:** Department of Surgery, Prince Charles Hospital, Merthyr Tydfil, Wales, UK

**Keywords:** Hernia, laparoscopic surgery, port site closure, port site

## Abstract

We have reported a novel technique for the closure of the ports after laparoscopic surgery. Using this technique all the ports are closed under vision, thus preventing port herniation.

## TECHNICAL TIP

We would like to report a novel method for the closure of the port site in laparoscopic surgery. There are usually 5 mm, 10 mm and 12 mm ports in laparoscopic surgery. The literature search reveals that the hernia through the port site can cause considerable morbidity in a post-operative patient especially requiring surgical intervention.[1] The use of bio-absorbable hernia plug has been recommended to prevent this hernias.[2] The non-bladed, radially dilating and conical blunt devices are also not without hazards of hernia.[3]

**Figure 1:**
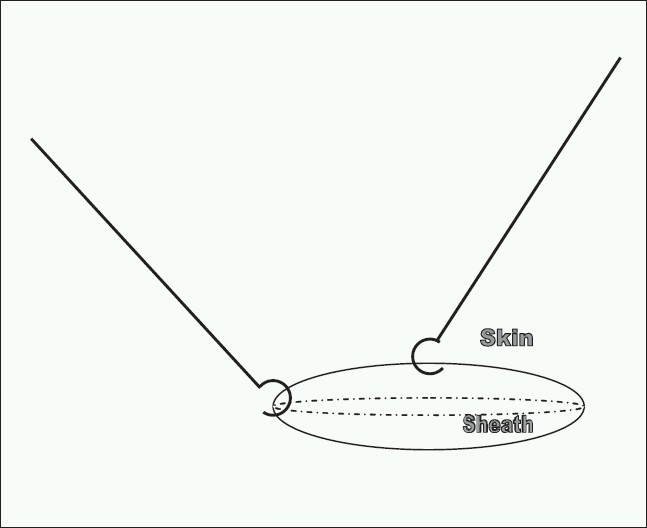
Taut sheath after insertion of the skin hook with Skin retraction by another skin hook

**Figure 2:**
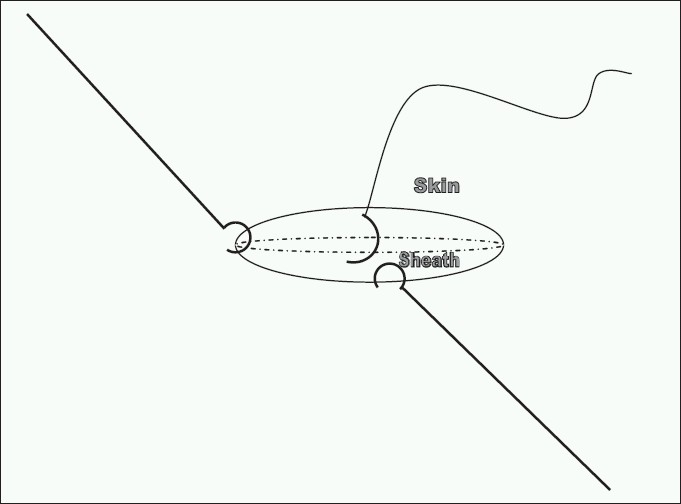
A stitch passed via one edge of the sheath and other edge now retracted with another hook

We routinely use Hassan's port for causing pneumo-peritoneum which is then closed under vision. The lateral 10 to 12 mm ports are closed using vicryl with the help of a skin hook and a retractor (small Langenbach right angled retractor or skin hook). Skin hook is inserted in the corner of the wound under the sheath. This can now be palpated with the finger very easily as the sheath is taut. This also causes approximation of the sheath which can now be accessed by using another retractor (right angled retractor/skin hook) above the sheath at 90 degrees to the hook (inserted under sheath) to retract skin and subcutaneous tissue [Figure 1]. The edge of the sheath is picked up with tooth forceps and a stitch is taken via one leaf of the sheath and the procedure is repeated by moving the retractor to the opposite edge to identify the other leaf of the sheath [Figure 2]. We have used this technique in our unit since last 7 years in over 120 laparoscopic bowel resection and fundoplications without single port site hernia.
